# Upper airway lengthening caused by weight increase in obstructive sleep apnea patients

**DOI:** 10.1186/s12931-020-01532-8

**Published:** 2020-10-19

**Authors:** Hongyi Lin, Huahui Xiong, Changjin Ji, Cunting Wang, Yong Li, Yunqiang An, Geng Li, Jianggui Guo, Xiaoqing Huang, Han Zhang, Hong Liu, Ting Li, Zheng Li, Junfang Xian, Yaqi Huang

**Affiliations:** 1 School of Biomedical Engineering, Capital Medical University, 10 Xitoutiao, Youanmenwai, Beijing, 100069 China; 2 Beijing Key Laboratory of Fundamental Research on Biomechanics in Clinical Application, Capital Medical University, Beijing, China; 3 Department of Radiology, Beijing Tongren Hospital, Capital Medical University, No 1 Dongjiaominxiang Street, Beijing, 100730 China

**Keywords:** Obstructive sleep apnea, Upper airway length, Body weight effect

## Abstract

**Background:**

The longer upper airway is more collapsible during sleep. This study aims to reveal relationships among upper airway length, weight, and obstructive sleep apnea (OSA), particularly to answer why the upper airway of OSA patients is longer than that of healthy people and why some obese people suffer from OSA while others do not.

**Methods:**

We perform head and neck MRI on male patients and controls, and measure > 20 morphological parameters, including several never before investigated, to quantify the effect of weight change on upper airway length.

**Results:**

The upper airway length is longer in patients and correlates strongly to body weight. Weight increase leads to significant fat infiltration in the tongue, causing the hyoid to move downward and lengthen the airway in patients. The apnea-hypopnea index (AHI) strongly correlates to airway length and tongue size. Surprisingly, a distance parameter h and angle β near the occipital bone both show significant differences between healthy males and patients due to their different head backward tilt angle, and strongly correlates with AHI. The contributions of downward hyoid movement and head tilt on airway lengthening are 67.4–80.5% and19.5–32.6%, respectively, in patients. The parapharyngeal fat pad also correlates strongly with AHI.

**Conclusions:**

The findings in this study reveal that the amount of body weight and distribution of deposited fat both affect airway length, and therefore OSA. Fat distribution plays a larger impact than the amount of weight, and is a better predictor of who among obese people are more prone to OSA.

## Introduction

Obstructive sleep apnea (OSA) is characterized by repeated collapse and obstruction of the upper airway (UA) during sleep [1]. UA geometry has significant effect on airflow characters, resistance, and obstruction [2, 3]. Previous studies have revealed that a longer airway has a considerably less negative closing pressure and is therefore more collapsible [4]. Morphological measurements do show that UA in patients is significantly longer compared with normal subjects [5, 6]. Studies also show a correlation between UA length and OSA severity [7, 8]. However, the mechanism of UA lengthening in OSA patients is unclear.

Obesity has a strong correlation with OSA [9–13]. It is estimated that 58% of moderate to severe OSA is due to obesity [14]. Because of increased fat deposition, overweight and obese individuals generally have a larger tongue [10, 15, 16] and more collapsible airway than normal-weight individuals [17–20]. From the observations that there is a high OSA prevalence in obese people and a long upper airway in many OSA patients, one may ask whether an increase in body weight can increase UA length, and therefore make UA more collapsible. There has currently not been any investigation focusing particularly on the relationship between UA length and weight change. A study observed that UA length decreased in OSA patients after 24 weeks of weight loss, but the reason remained unclear [21]. A quantitative investigation about the different effect of body weight on UA length between normal people and OSA patients may solve the puzzle: why do some people with obesity suffer from OSA while others do not?

In this study, we will test the hypothesis: both the amount of body weight and distributions of deposited fat in the head and neck can affect airway length, but the fat distribution dominates the change in UA length and determines who among obese people are more prone to OSA.

## Methods

### Subjects

Chinese male candidates were recruited through public notice boards and determined through overnight polysomnography monitoring. Eighteen patients with apnea-hypopnea index (AHI) > 15 (AHI = 43.59 ± 17.57), who may exhibit typical OSA characteristics, participated in this study. Twenty healthy subjects were selected by a careful evaluation based on their basic information and survey results. A qualified candidate should have no symptoms such as snoring, sleep apnea, and daytime somnolence. We also performed polysomnography tests for six candidates, 30% of all healthy subjects, to ensure that their AHI < 5. We did not match the body mass index (BMI) between the two groups in order to better observe the effect of weight on UA length in a larger BMI range. We also formed two BMI matching groups with weight 69–93 kg, which could maximize the size of each group to include 14 OSA patients and 13 healthy subjects, to observe the similarities and differences in morphological parameters between OSA patients and healthy people with similar BMI. The data were analyzed with age as a covariate to account for the effect of age. Table 1 summarizes the characteristics of the subjects. Nobody had heart failure, renal failure, tonsillectomy, or uvulopalatopharyngoplasty. Nobody had been previously treated for OSA. The ethics committee of Capital Medical University approved the study (2013SY67), and all subjects signed the informed consent.

**Table 1:** Characteristics of male OSA patients and healthy subjects

	No BMI matching			BMI matching		
	OSA patients (*n* = 18)	Healthy subjects (*n* = 20)	*P* value	OSA patients (*n* = 14)	Healthy subjects (*n* = 13)	*P* value*
Age, y	48.11 ± 12.56	37 ± 13.73	0.015	51 ± 12.16	36.62 ± 12.63	–
Height, cm	174.33 ± 7.09	172.47 ± 5.84	0.237	171.23 ± 5.25	172.54 ± 6.01	0.376
Weight, kg	83.36 ± 13.5	70.16 ± 9.15	0.001	76.64 ± 5.9	75.08 ± 5.51	0.352
BMI, kg.m^−2^	27.35 ± 3.5	23.63 ± 3.22	0.002	26.16 ± 1.93	25.25 ± 1.84	0.784

Data are presented as mean ± SD. *OSA* obstructive sleep apnea, *BMI* body mass index; *: *P*-value after adjustment for age

### Magnetic resonance imaging (MRI)

Using a spoiled gradient echo sequence described in details in the references [22–24], we performed axial and sagittal scanning in a 3.0 T MRI scanner (Signa HDxt, General Electric, USA) to obtain images from the nasal cavity to the laryngeal prominence. Subjects were awake and lying supine. They were told not to swallow during scanning. Because obese people had a larger head backward tilt angle when lying supine than subjects with normal weight, in order to observe the effect of head tilt angle on UA geometry, we also performed MRI under three states: lying supine, with partial chin elevation, and with a maximum chin elevation in healthy subjects.

### Reconstruction of tissue structures and measurement of morphological parameters

**Fig. 1:**
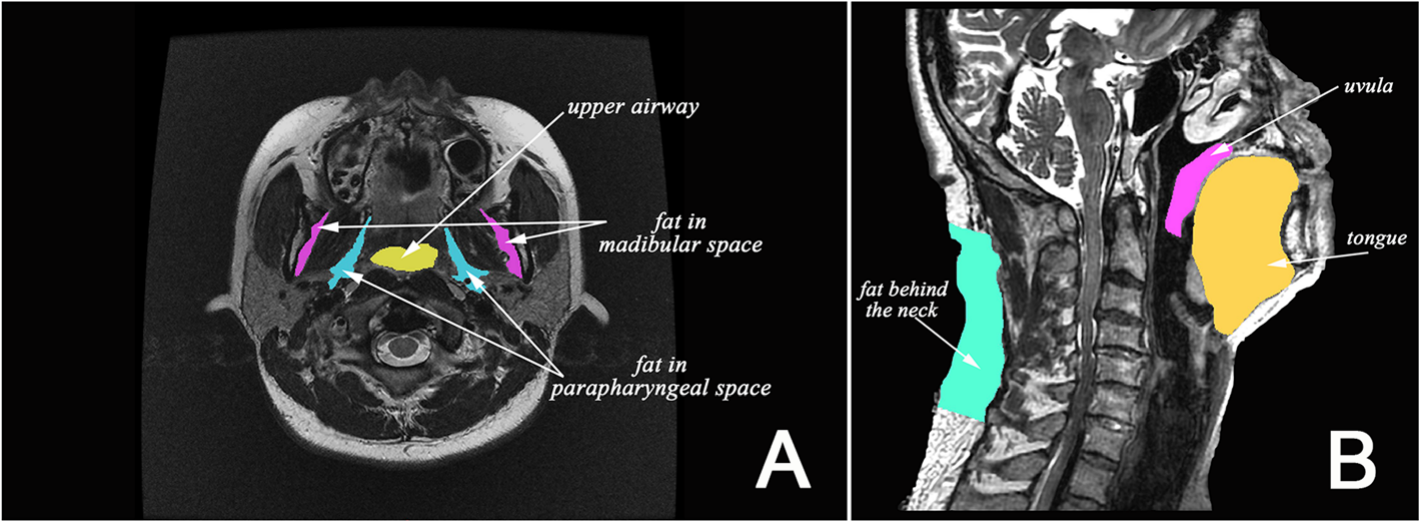
Segmentation of partial tissues

We segmented tissues from the images manually, reconstructed the three-dimensional structures of UA, tongue, fats in the mandibular space and parapharyngeal space, fat behind the neck, and posterior cervical soft tissue using Mimics (Materialise Inc., Leuven, Belgium), and measured their morphological parameters including the tissue volume, cross-sectional area, length, angle, and relative positions. Figure 1 diagrams some of the tissues measured in this study.

**Fig. 2:**
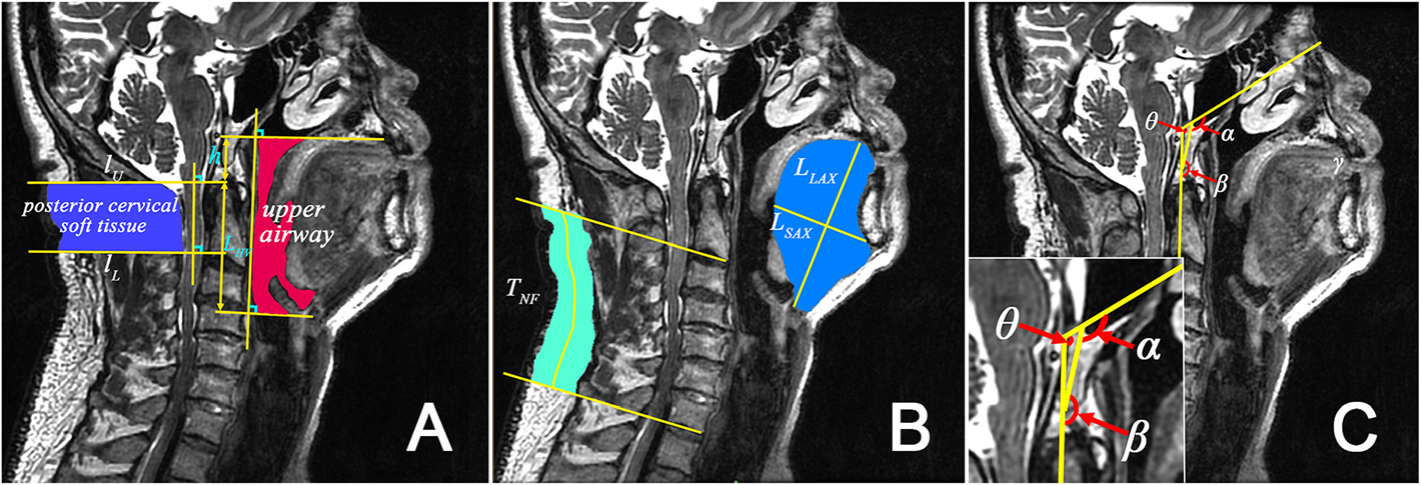
Definitions of main parameters on the midsagittal plane. In **a**, the two lines drawn from the hard palate, near the top of the oral cavity, and the hyoid, which are also normal to the posterior wall, define the two ends of the upper airway. The parameter h, a part of the upper airway length, is the distance from the upper edge of the second cervical vertebra to the hard palate plane. The average thickness of the posterior cervical soft tissue T_ST_ is calculated from the area surrounded by the three lines and skin dividing the distance between the two parallel lines passing through the upper and lower edges of the second cervical vertebra. In **b**, the long axis of the tongue L_LAX_ starts from the central point of the hyoid bone and ends at a point farthest from the hyoid on the tongue surface, and the short axis L_SAX_ is the perpendicular bisector of L_LAX_ ending on the tongue surfaces. The average thickness of the posterior cervical fat T_NF_ is calculated by the area of the fat beneath the skin, from the bottoms of the second to the sixth cervical vertebrae, dividing the length of the central line. In **c**, The angles α and β represent the relative positions among the occipital bone, cervical vertebra, and nasion: β is the angle between the posterior wall of the upper airway and the occipital bone line drawn from the sellar to the tip of the clivus, and α is the angle between the occipital bone line and the line drawn from the nasion to the sellar. θ is the angle between the posterior wall of the upper airway and the line drawn from the nasion to the sellar. The lower left corner is a magnification of the local region defining these three angles

Figure 2 defines some structural geometrical parameters on the midsagittal plane. The two lines defining the ends of UA in Fig. 2a represent the hard palate plane and the hyoid bone plane in the three-dimensional structure of the head and neck, respectively. A key parameter h proposed in this study, which is the distance from the upper edge of the second cervical vertebra to the hard palate plane, has never been investigated before. L_HV_ is the distance between the top of the second cervical vertebra and the hyoid bone plane. T_ST_ is the average thickness of the posterior cervical soft tissue. Figure 2b shows the long axis (L_LAX_) and short axis (L_SAX_) of the tongue, and also the average thickness of the posterior cervical fat (T_NF_). The angles β and θ, which have also never been studied previously, together with the angle α are defined in Fig. 2c. The angle θ = β + α-180^o^ can describe the level of head tilt. In addition, we also measure the neck circumference (C_N_) and a non-head and neck parameter, the waist circumference (C_W_), using a measuring tape.

### Quantitative evaluations of weight effects on upper airway length

UA length L_UA_ is the sum of h and L_HV_. Head tilt can affect not only h but also L_HV_. We express L_UA_ and L_HV_ as a linear function of the level of head tilt, or the angle θ: L_UA_ = a_1_θ + b_1_ and L_HV_ = a_2_θ + b_2_. From the two equations, a Δθ increase in angle will increase the UA length by ΔL_UA_ = a_1_Δθ and simultaneously result in an upward movement of the hyoid bone plane by an amount |ΔL_HV_| = |a_2_|Δθ in these healthy subjects. The hyoid position in OSA patients should be lower than in healthy subjects when there was no difference in the head tilt angle between the two groups. If the amount of downward movement of the hyoid, denoted by ΔL_hyoid_, in OSA patients is similar to the amount of upward movement |ΔL_HV_| caused by an angle change Δθ^*^ in healthy subjects, we can estimate the hyoid moving down caused by the large weight in OSA patients using ΔL_hyoid_ = |ΔL_HV_| when Δθ^=^Δθ^*^ or the expression ΔL_hyoid_ = |a_2_|Δθ^*^. Therefore, the total increase in UA length resulted from head tilt and hyoid movement for patients is ΔL_OSA_ = ΔL_UA_ + ΔL_hyoid_ = a_1_Δθ + |a_2_|Δθ^*^. The contribution of the increase caused by Δθ to the total increase in UA length in patients is ΔL_UA_/ΔL_OSA_ = Δθ/(Δθ + |a_2_/a_1_|Δθ^*^), and the contribution of the downward movement of the hyoid is ΔL_hyoid_/ΔL_OSA_ = |a_2_/a_1_|Δθ^*^/(Δθ + |a_2_/a_1_|Δθ^*^). Measuring L_UA_ and L_HV_ at different angles θ caused by different levels of head tilt in healthy subjects, we can determine the parameters a_1_ and a_2_ by fitting the data to the equations L_UA_ = a_1_θ + b_1_ and L_HV_ = a_2_θ + b_2_. The parameter Δθ^*^ can be determined by comparing the position of the hyoid bone plane between the OSA patients and healthy subjects, which will be addressed in detail in the Results section.

### Statistical analysis

All morphological data measured from structures of OSA patients and healthy subjects were analyzed using the software SPSS (SPSS Inc., Chicago, USA). Independent-sample t test was used in data comparisons between the patients and controls. Covariance analysis was used to adjust for age in the comparison between the patient and control groups with BMI matching. The value *p* < 0.05 was considered as statistically significant. Correlation analysis was used to test relationships between measured parameters.

## Results

### Statistical analysis of morphological parameters

**Fig. 3:**
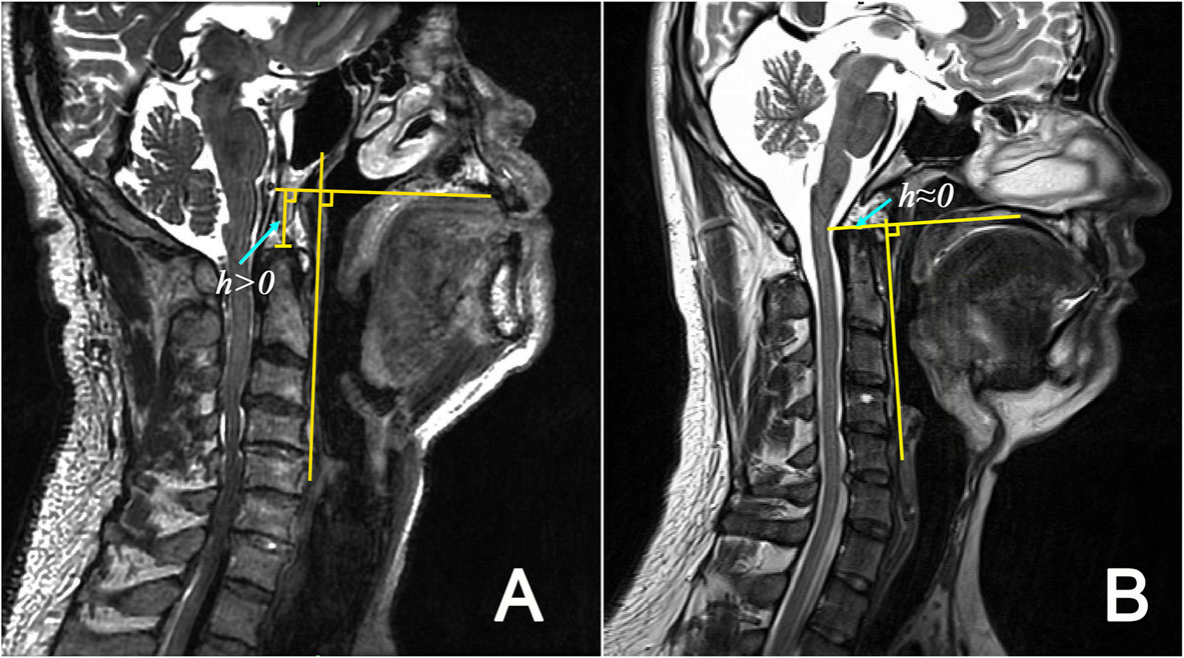
The relative position between the upper edge of the second cervical vertebra and the hard palate plane, **a** for obstructive sleep apnea patients and **b** for healthy subjects

Table 2 lists the measured results of the histomorphological parameters. Comparing OSA patients to healthy subjects, there are significant differences in h, β, and T_ST_. As shown in Fig. 3, the upper edge of the second cervical vertebra in OSA patients is generally under the hard palate plane (h > 0) while it is generally above the hard palate plane (h < 0) or near the hard palate plane in healthy subjects. There is a significant difference in h between healthy subjects and OSA patients whether BMI is matched or not (both *p* < 0.001). The mean value of β for OSA patients is much larger than in healthy groups with and without BMI matching (both *p* < 0.001). The mean value of α of OSA patients is smaller than in healthy subjects with BMI matching (*p* = 0.042) or without (*p* = 0.027), which is consistent with the statistical results of Neelapu et al. [25] A correlation analysis for all 38 subjects in healthy and patient groups shows a negative correlation between the angles α and β (*r* = 0.465, *p* = 0.004).

**Table 2:** Histomorphological measurement results in male OSA patients and healthy subjects

	No BMI matching			BMI matching		
	OSA patients (*n* = 18)	Healthy subjects (*n* = 20)	*P* value	OSA Patients (*n* = 14)	Healthy subjects (*n* = 13)	*P* value*
Length of the upper airway (L_UA_), mm	82.79 ± 6.02	68.8 ± 6.83	< 0.001	81.99 ± 5.88	70.19 ± 6.41	0.001
Upper airway volume, cm^3^	12.71 ± 5.25	10.67 ± 6.3	0.285	11.67 ± 3.91	11.71 ± 7.2	0.66
Narrowest cross-sectional area of the upper airway (S_NUA_), mm^2^	25.1 ± 19.41	65.32 ± 41.32	0.001	22 ± 15.86	64.25 ± 36.55	0.001
Average cross-sectional area of the upper airway (S_AUA_), mm^2^	147.01 ± 58.77	167.12 ± 74.89	0.361	135.73 ± 45.61	177.82 ± 83.27	0.099
Fat volume in parapharyngeal space (V_PF_), cm^3^	11.15 ± 4.2	6.25 ± 2.73	< 0.001	10.25 ± 3.12	7.17 ± 2.87	0.045
Fat volume in mandibular space (V_MF_), cm^3^	3.65 ± 1.72	1.73 ± 0.75	< 0.001	3.71 ± 1.51	1.58 ± 0.74	0.002
Thickness of posterior cervical fat (T_NF_), mm	17 ± 5.43	13.22 ± 5.8	0.037	15.67 ± 4	15.18 ± 4.84	0.623
Fat area in parapharyngeal space in the neck plane with the narrowest upper airway (S_NPF_), mm^2^	279.37 ± 130.91	176.2 ± 116.17	0.015	262.07 ± 139.68	197.96 ± 116.38	0.072
Thickness of soft tissue behind neck (T_ST_), mm	63.97 ± 7.39	50.03 ± 6.84	< 0.001	62.29 ± 5.26	52.65 ± 5.05	< 0.001
Tongue volume (V_TG_), cm^3^	138.23 ± 13.79	119.21 ± 12.64	< 0.001	136.41 ± 10.4	122.01 ± 13.15	< 0.001
Length of the long axis of tongue (L_LAX_), mm	82.65 ± 7.76	68.33 ± 5.81	< 0.001	81.31 ± 6.87	69.94 ± 6.13	0.002
Length of the short axis of tongue (L_SAX_), mm	48.13 ± 4.48	50.73 ± 6.17	0.151	48.25 ± 4.38	51.38 ± 5.18	0.588
Ratio of the long to short axis of tongue,	1.73 ± 0.21	1.37 ± 0.26	< 0.001	1.7 ± 0.23	1.38 ± 0.24	0.027
Distance from the top of the second cervical vertebra to hyoid bone plane (L_HV_), mm	70.09 ± 6.4	69.37 ± 8.2	0.764	71.45 ± 6.37	67.96 ± 8.23	0.822
Distance from the top of the second cervical vertebra to hard palate plane (h), mm	12.42 ± 7.71	−0.76 ± 6.06	< 0.001	10.31 ± 5.9	1.11 ± 5.22	0.001
α, °	140.04 ± 6.08	144.67 ± 6.08	0.027	139.73 ± 6.8	145.24 ± 6.24	0.029
β, °	149.05 ± 8.54	134.55 ± 6.27	< 0.001	147.11 ± 7.11	135.85 ± 5.55	< 0.001
θ, °	109.04 ± 10.67	99.22 ± 4.82	0.002	106.62 ± 9.99	101.09 ± 4.52	0.23
Neck circumference, (C_N_), cm	40.76 ± 2.14	36.56 ± 1.91	< 0.001	40.25 ± 1.44	36.87 ± 2.20	< 0.001
Waist circumference, (C_W_), cm	97.36 ± 9.19	86.08 ± 8.77	0.001	94.06 ± 5.49	89.92 ± 7.34	0.133

Data are presented as mean ± SD. *OSA* obstructive sleep apnea, *BMI* body mass index; *: *P*-value after adjustment for age

**Fig. 4:**
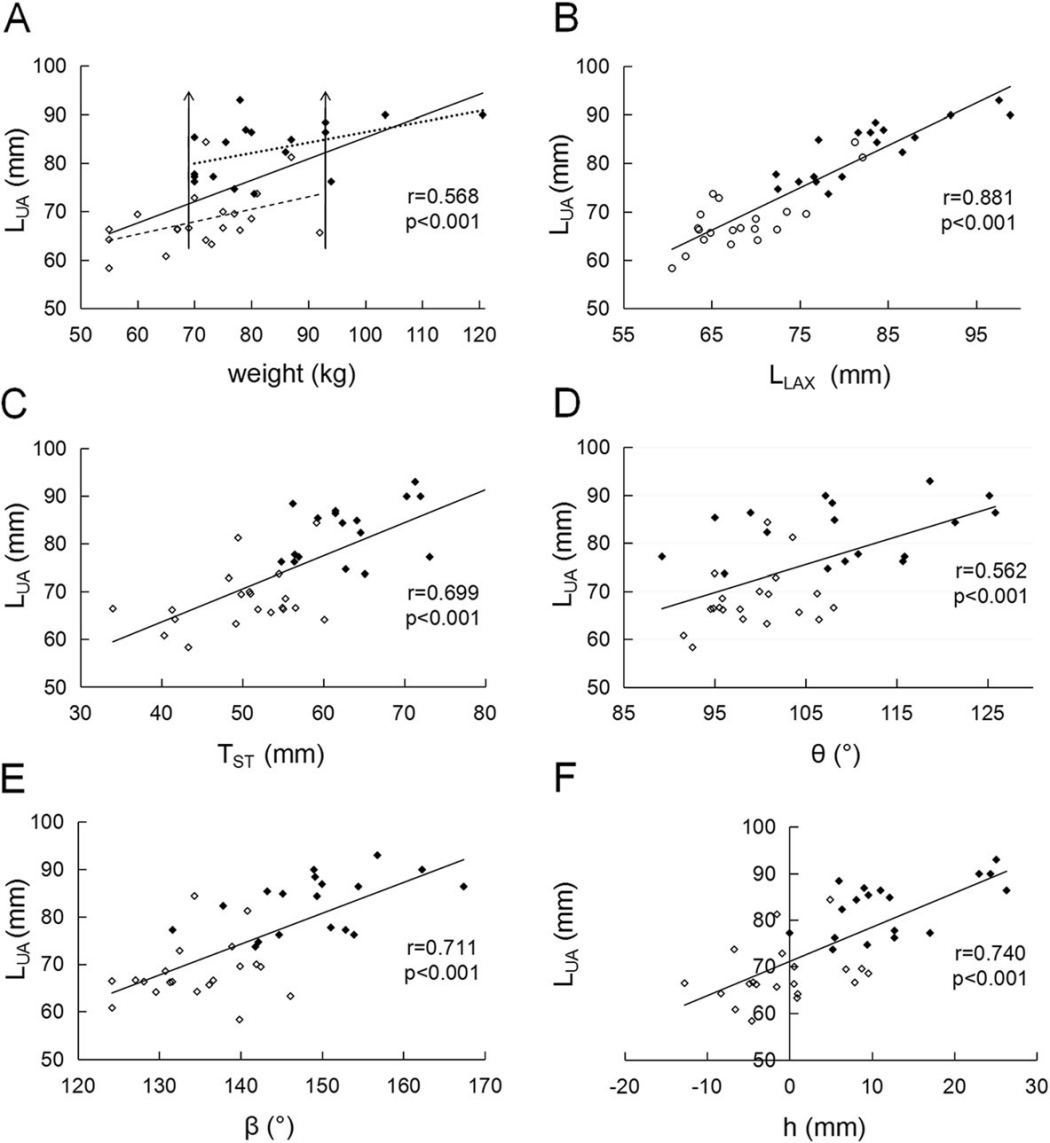
Correlations of the upper airway length L_UA_ with **a** the body weight W, **b** the length of the long axis of tongue L_LAX_, **c** the thickness of soft tissue behind the neck T_ST_, **d** the angle θ, **e** the angle β, and **f** the distance from the top of second cervical vertebra to hard palate plane h in all subjects. Solid symbols represent obstructive sleep apnea patients and hollow symbols are healthy subjects. Solid lines are the fitting results for data from all subjects. The upper and lower dashed lines in subfigure **a** are fitting results for patients and healthy subjects, respectively. The two vertical lines indicate the weight range with body mass index matching in patient and healthy groups

**Fig. 5:**
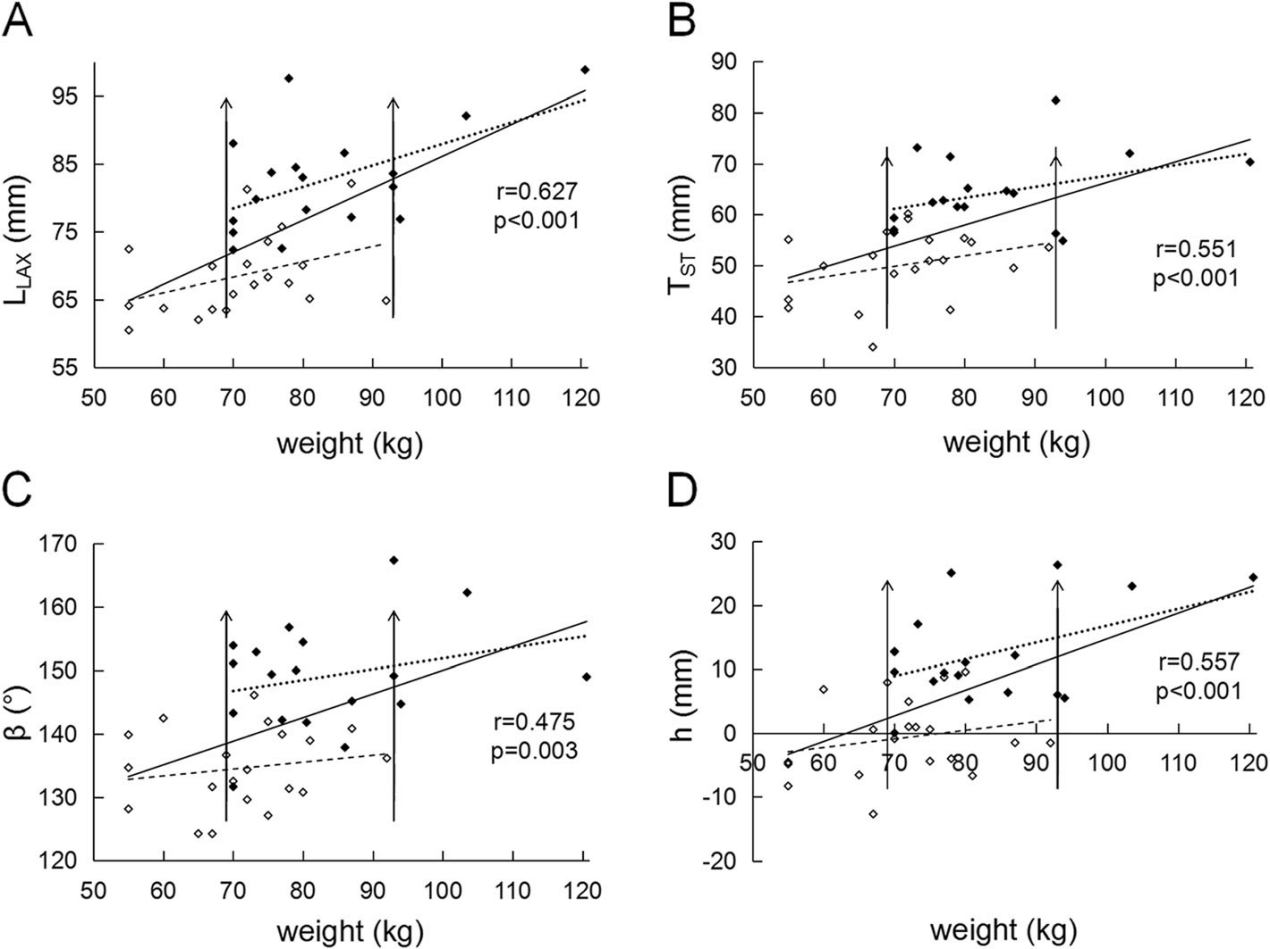
Correlations of the body weight W with **a** the length of the long axis of tongue L_LAX_, **b** the thickness of soft tissue behind the neck T_ST_, **c** the angle β, and **d** the distance from the top of second cervical vertebra to hard palate plane h in all subjects. Solid symbols represent obstructive sleep apnea patients and hollow symbols are healthy subjects. Solid lines are the fitting results for data from all subjects. The upper and lower dashed lines are the fitting results for patients and healthy subjects, respectively. The two vertical lines indicate the weight range with body mass index matching in patient and healthy groups

As shown in Table 2, OSA patients have longer UA than healthy subjects whether BMI is matched or not (both *p* < 0.001). UA length L_UA_ has strong correlation with BMI (*r* = 0.573, *p* < 0.001), C_N_ (*r* = 0.674, *p* < 0.001), and C_w_ (*r* = 0.861, *p* < 0.001) respectively, suggesting that body weight (W) plays an important role in L_UA_ change. Figure 4 shows that W, L_LAX_, T_ST_, θ, β, and h are all strongly correlated with L_UA_ (*p* < 0.001). L_UA_ also shows a weak negative correlation with α (*r* = − 0.324, *p* = 0.051). Figure 5 shows that h (*p* < 0.001), T_ST_ (*p* < 0.001), β (*p* = 0.003), and L_LAX_ (*p* < 0.001) are strongly correlated with W. One can see from Table 2, Fig. 4a, and Fig. 5 that although the body weight in BMI matching patient and control groups is similar (*p* = 0.765), there are still significant differences in L_UA_, h, T_ST_, β, L_LAX_ and C_N_ between the two groups (all *p* < 0.001), but α is unrelated to W (*r* = − 0.107, *p* = 0.528).

Interestingly, the slopes of the two dashed lines for OSA patients and healthy subjects in Figs. 4a and 5 are almost the same, suggesting that the variation of these parameters with body weight are the same in the two groups. However, there is a distance between the two lines, showing differences in these morphological parameters. We can infer that the weight effect on morphological parameters such as UA length is not only from the increased total body weight, but also from the distribution of weight gained.

Larger weight can lead to longer UA in both healthy subjects and OSA patients. However, from the BMI matching data in Table 2, we can see that the parameters C_N_ and T_ST_ in the patient group are significantly larger than in the healthy group, which leads to larger θ and β. In order to evaluate the contributions of the head tilt angle on UA morphology, we use MRI results obtained from 19 healthy subjects with partial and a maximum chin elevations. The results indicate that, with the increase in the head tilt angle, the position of the hard palate and the hyoid moved upward relative to the cervical vertebra, resulting in an increase in h and a decrease in L_HV_. By fitting the data at different head tilt angles, we obtain a_1_ = 0.457 and a_2_ = − 0.946 in the equations given in the Methods section, and then we obtain |a_2_/a_1_| = 2.07.

The data without BMI matching in Table 2 shows that θ in the OSA patient group was 10° larger than in the healthy group, while the distance L_HV_ was almost the same. This indicates that the hyoid position in OSA patients should be lower than in healthy subjects when there was no difference in the head tilt angle between the two groups, and the amount of downward movement of the hyoid in OSA patients should be similar to the amount of upward movement caused by the 10° change in θ in healthy subjects. Thus, the parameter Δθ^*^ = 10°, and the large weight leads to the hyoid moving down by ΔL_hyoid_ = 10|a_2_| in OSA patients. Therefore, in addition to the effect of increased head tilt angle while gaining weight, the downward movement of the hyoid bone is another major reason of UA lengthening in patients. The contribution of Δθ is ΔL_UA_/ΔL_OSA_ = Δθ/(Δθ + 20.7), and the contribution of the downward movement of the hyoid is ΔL_hyoid_ /ΔL_OSA_ = 20.7/(Δθ + 20.7). Therefore, we can estimate that when the head tilt angle in OSA patients is 5–10° larger than in healthy people, head tilt contributes about 19.5–32.6% or roughly 1/5–1/3, and the hyoid moving down contributes 67.4–80.5% or roughly 2/3–4/5, to the increase in UA length in OSA patients.

The data for BMI matching in Table 2 indicates that the tongue volume V_TG_ of OSA patients is significantly larger than in healthy subjects (*p* = 0.007). This suggests that although the two groups have similar body weight, there is more fat in the tongue, and therefore a larger tongue, for patients. Due to the limited space in the anterior-posterior direction, there is no significant difference in L_SAX_ between patients and healthy people (*p* = 0.114). Therefore, an enlarged tongue will lead directly to an increase in L_LAX_ (*p* < 0.001) and a downward movement of the hyoid bone. This is consistent with the observations of Chi et al. [16] Although there are significant differences in the narrowest cross-sectional area of UA (S_NUA_) between OSA patients and healthy subjects (*p* = 0.001), there are no significant differences in the average cross-sectional area of UA (S_AUA_) (*p* = 0.361) and UA volume (*p* = 0.285). This is due to stronger genioglossus reactions in patients under waking condition [26–28], which can keep the size of UA cross-section steady to maintain normal breathing.

Compared with healthy subjects, OSA patients have larger neck circumference C_N_, BMI matched or not, and obvious fat depositions in some neck regions. In the OSA patients, the volume of fat in the parapharyngeal space V_PF_ and mandibular space V_MF_ both increase significantly compared to healthy subjects, BMI matched or not. The thickness of posterior cervical fat T_NF_ and waist circumference C_W_ increase with weight increase. The data for BMI matching showed that for similar body weight, there is no significant difference in T_NF_ or C_W_ between patients and healthy subjects.

### Correlation analysis between morphological parameters and AHI

**Fig. 6:**
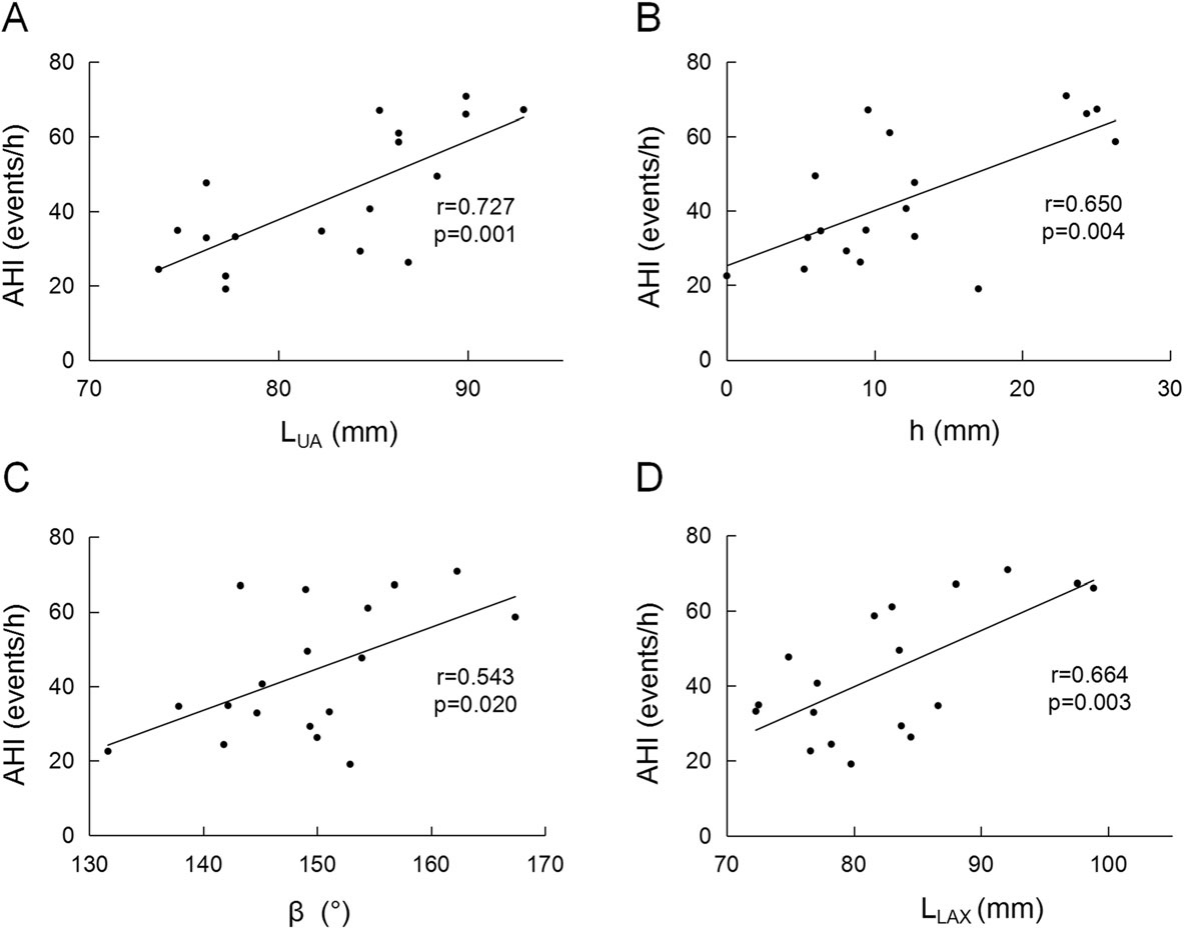
The correlations between apnea-hypopnea index (AHI) and **a** upper airway length L_UA_, **b** distance from hard palate plane to the top of second cervical spine h, **c** angle β, and **d** length of the long axial of the tongue L_LAX_ in obstructive sleep apnea patients

The correlation analysis for the data of 18 OSA patients shows that AHI is strongly correlated with UA length (Fig. 6a), h (Fig. 6b), β (Fig. 6c), the fat area in parapharyngeal space in the neck plane with the narrowest UA (S_NPF_) (*r* = 0.478, *p* = 0.045), and waist circumference (*r* = 0.625, *p* = 0.013). Although S_NUA_ and C_N_ are significantly different between patients and healthy subjects, there is no significant correlation between AHI and S_NUA_ or C_N_. There is no significant correlation between AHI and V_PF_, V_MF_, T_NF_ in OSA patients, but AHI shows strong positive correlations with V_TG_ (*r* = 0.569, *p* = 0.017), and L_LAX_ (Fig. 6d).

## Discussion

This is the first study to address quantitatively the effect of body weight on UA length, and therefore UA collapsibility. By systematic morphological measurements on tissues surrounding UA and quantitative analyses, the study reveals the roles played by body weight in UA lengthening and obstruction. In addition to confirming that UA of OSA patients is much longer than in healthy subjects, and that L_UA_ is positively correlated with AHI as observed in some studies [8], we find the significant impact of body weight on L_UA_ and the mechanism of UA lengthening in obese people, suggesting that weight gain increases L_UA_ and the risk of UA collapse during sleep. In a study involving 24 weeks of weight loss, the investigators observed a decrease in the distance between the hard palate and hyoid bone in OSA patients [21]. This reflects our own observations, which show that weight change will lead to L_UA_ change in the same direction.

It sounds amazing that weight increase can make UA longer. A clarification of its mechanism is particularly important for the treatment of OSA. From our hypothesis and findings, there are two points that merit special attention. One is the impact of the total weight increase, and the other is the impact of the regional distribution of the increased weight. Our results show that the distance h increases with weight increase in both OSA patients and healthy people, but the increment is different after weight matching. We can explain this as follows: Table 2 shows that T_ST_ in OSA patients is significantly larger than in healthy subjects, BMI matched or not. This increase in thickness will tilt the head farther back, increasing β, which then leads to an increase in the distance h and therefore lengthens UA. Another reason for airway lengthening is that the pulling and squeezing on the surrounding tissues of the airway due to fat deposition, especially the expansion of tongue volume, will push the hyoid down and increase L_HV_. Obesity leads to the increase of fat content in the genioglossal muscle [20], which can increase tongue size. Our results show that the tongue volume V_TG_ in OSA patients is significantly larger than in healthy subjects. The head tilt angle and hyoid downward displacement are both related to the fat increase. This can explain morphologically why not all obese people suffer from OSA. We found that OSA patients have a 5–10° larger head tilt angle than in healthy people due to their large T_ST_, and the contributions of hyoid downward movement and head tilt on the change in L_UA_ are 67.4–80.5% and 19.5–32.6%, respectively. Although the amount of weight and the distribution of deposited fat will both affect L_UA_, Fig. 4a shows that the impact of fat distribution is larger than the increased amount of weight, which proves our hypothesis.

We grouped subjects not only with matched BMI between OSA patients and healthy subjects, but also without BMI matching to extend the weight range. From the BMI matching groups, we can observe the difference of fat distribution in the head and neck between the patient and healthy groups and analyze its impacts on respiratory functions. From the groups without BMI matching, we can observe some important phenomena over a large weight range, such as the changes in the parameters h, β, V_TG_, L_LAX_ and L_UA_ with weight. Our study shows that fat distribution plays a key role in evaluating the changes in UA length. Because BMI is an overall parameter that cannot provide any distribution information, it is therefore hard to effectively evaluate the impact of weight increase on the occurrence of OSA for an individual patient using BMI only.

The fat in the head and neck accumulates when weight increases in OSA patients. Some previous studies have found that parapharyngeal fat pads can narrow UA [29], while other data do not show such effects [30]. Our results show significant differences in V_PF_ and V_MF_ between OSA patients and healthy subjects, and S_NPF_ correlates with AHI. This suggests that fat accumulated at the parapharyngeal and mandibular areas will directly compress UA. Therefore, obesity has a double effect on the morphology of UA in OSA patients. On the one hand, it will lengthen UA. On the other hand, the increase of fat around the pharynx will squeeze and narrow UA when most of the dilator muscle activations are lost during sleep. Both factors make airway collapse and obstruction more likely during sleep in OSA patients. Our results show that the parameter S_NUA_ in OSA patients is significantly smaller than in healthy subjects, but there is no correlation between S_NUA_ and AHI. This is not surprising because the images were taken while awake rather than sleeping, and therefore the parameter S_NUA_ only reflects the narrowest caliber of UA while awake. Because dilator muscle activities can be very different among patients under waking condition for maintaining normal breathing, the distribution of the narrowed size of UA obtained under such a condition can be very different from that during sleep, when most of dilator muscle activities are lost.

We should mention that the number of patients included in the study is still small and all subjects in this study are male. The population of males with OSA is much higher than females [31], and we design, as the first step, the comparisons of morphological parameters between male healthy subjects and patients to study the effects of body weight on the airway length and therefore on OSA. Further investigations are needed to see whether the important conclusions of this study can be extend to females. Another limitation is that our patient group does not include mild OSA patients with AHI between 5 and 15. We only measured and analyzed data for moderate and severe OSA patients with AHI > 15 in this study. In addition, the subjects participated in this study are Chinese. We do not know whether the findings of this study can be applied to other population groups.

## Conclusions

The important findings in this study prove our hypothesis, and reveal that although the amount of body weight and the distribution of deposited fat both affect airway length, and therefore OSA, the impact of fat distribution is larger than that of weight increase, and is a better predictor of who among obese people are more prone to OSA. The findings in this study can lead to a better understanding of OSA mechanism and will be of great significance in developing effective treatments for OSA.

## Data Availability

The datasets used and/or analysed during the current study are available from the corresponding author on reasonable request.

## Abbreviations

AHI: The apnea-hypopnea index
BMI: The body mass index
C_N_: The neck circumference
C_W_: The waist circumference
h: The distance from the upper edge of the second cervical vertebra to the hard palate plane
L_HV_: The distance between the top of the second cervical vertebra to the hyoid bone plane
L_LAX_: The long axis of the tongue
L_SAX_: The short axis of the tongue
L_UA_: The upper airway length
MRI: Magnetic resonance imaging
OSA: Obstructive sleep apnea
S_AUA_: The average cross-sectional area of the upper airway
S_NPF_: The area of fat in parapharyngeal space in the neck plane with the narrowest upper airway
S_NUA_: The narrowest cross-sectional area of the upper airway
T_NF_: The average thickness of the posterior cervical fat
T_ST_: The average thickness of the posterior cervical soft tissue
UA: The upper airway
V_MF_: The volume of fat in the mandibular space
V_PF_: The volume of fat in the parapharyngeal space
V_TG_: The tongue volume
W: The body weight
α: The angle between the occipital bone line and the line drawn from the nasion to the sellar
β: The angle between the posterior wall of the upper airway and the occipital bone line drawn from the sellar to the tip of the clivus
θ: The angle between the posterior wall of the upper airway and the line drawn from the nasion to the sellar
ΔL_hyoid_: The downward moving distance of the hyoid bone
ΔL_OSA_: The total increase in the upper airway length resulted from head tilt and hyoid movement
ΔL_UA_: The increase in the upper airway length
Δθ: The increase in the angle θ

## References

[1] Malhotra A, White DP (2002). Obstructive sleep apnoea. Lancet..

[2] Wu H, Wang M, Wang J, An Y, Wang H, Huang Y (2019). Direct visualizations of air flow in the human upper airway using in-vitro models. Sci China Life Sci.

[3] Wang S, Zhang H, Huang X, et al. A pilot study in male patients to show the effects of natural fluid shift on the severity of obstructive sleep apnea. Sleep Breath. 2020. 10.1007/s11325-020-02044-x.

[4] Malhotra A, Huang Y, Fogel RB (2002). The male predisposition to pharyngeal collapse: importance of airway length. Am J Respir Crit Care Med.

[5] Finkelstein Y, Wolf L, Nachmani A (2014). Velopharyngeal anatomy in patients with obstructive sleep apnea versus normal subjects. J Oral Maxillofac Surg.

[6] Dentino K, Ganjawalla K, Inverso G, Mulliken JB, Padwa BL (2015). Upper airway length is predictive of obstructive sleep apnea in syndromic craniosynostosis. J Oral Maxillofac Surg.

[7] Susarla SM, Abramson ZR, Dodson TB, Kaban LB (2010). Cephalometric measurement of upper airway length correlates with the presence and severity of obstructive sleep apnea. J Oral Maxillofac Surg.

[8] Segal Y, Malhotra A, Pillar G (2008). Upper airway length may be associated with the severity of obstructive sleep apnea syndrome. Sleep Breath.

[9] Sutherland K, Lee RW, Cistulli PA (2012). Obesity and craniofacial structure as risk factors for obstructive sleep apnoea: impact of ethnicity. Respirology.

[10] Ito E, Tsuiki S, Maeda K, Okajima I, Inoue Y (2016). Oropharyngeal crowding closely relates to aggravation of OSA. Chest.

[11] Tuomilehto H, Seppä J, Uusitupa M (2012). Obesity and obstructive sleep apnea-clinical significance of weight loss. Sleep Med Rev.

[12] Newman AB, Foster G, Givelber R, Nieto FJ, Redline S, Young T (2005). Progression and regression of sleep-disordered breathing with changes in weight: the sleep heart health study. Arch Intern Med.

[13] Peppard PE, Young T, Palta M, Dempsey J, Skatrud J (2000). Longitudinal study of moderate weight change and sleep-disordered breathing. JAMA.

[14] Young T, Peppard PE, Taheri S (2005). Excess weight and sleep-disordered breathing. J Appl Physiol (1985).

[15] Schellenberg JB, Maislin G, Schwab RJ (2000). Physical findings and the risk for obstructive sleep apnea, the importance of oropharyngeal structures. Am J Respir Crit Care Med.

[16] Chi L, Comyn FL, Mitra N (2011). Identification of craniofacial risk factors for obstructive sleep apnoea using three-dimensional MRI. Eur Respir J.

[17] McGinley BM, Schwartz AR, Schneider H, Kirkness JP, Smith PL, Patil SP (2008). Upper airway neuromuscular compensation during sleep is defective in obstructive sleep apnea. J Appl Physiol (1985).

[18] Pahkala R, Seppä J, Ikonen A, Smirnov G, Tuomilehto H (2014). The impact of pharyngeal fat tissue on the pathogenesis of obstructive sleep apnea. Sleep Breath.

[19] Li Y, Lin N, Ye J, Chang Q, Han D, Sperry A (2012). Upper airway fat tissue distribution in subjects with obstructive sleep apnea and its effect on retropalatal mechanical loads. Respir Care.

[20] Kim AM, Keenan BT, Jackson N (2014). Tongue fat and its relationship to obstructive sleep apnea. Sleep.

[21] Sutherland K, Lee RW, Phillips CL (2011). Effect of weight loss on upper airway size and facial fat in men with obstructive sleep apnoea. Thorax.

[22] An Y, Li Y, Liu Z (2015). Effects of fluid shift on upper airway patency and neck circumference in normal-weight subjects. Sleep Med.

[23] Lin H, Wang C, Zhang H (2019). Threshold of the upper airway cross-section for hypopnea onset during sleep and its identification under waking condition. Resp Res.

[24] An Y, Ji C, Li Y, Wang J, Zhang X, Huang Y (2017). In vivo measurements of human neck skin elasticity using MRI and finite element modeling. Med Phys.

[25] Neelapu BC, Kharbanda OP, Sardana HK (2017). Craniofacial and upper airway morphology in adult obstructive sleep apnea patients: a systematic review and meta-analysis of cephalometric studies. Sleep Med Rev.

[26] Mezzanotte WS, Tangel DJ, White DP (1992). Waking genioglossal electromyogram in sleep apnea patients versus normal controls (a neuromuscular compensatory mechanism). J Clin Invest.

[27] Pierce R, White D, Malhotra A (2007). Upper airway collapsibility, dilator muscle activation and resistance in sleep apnoea. Eur Respir J.

[28] Carberry JC, Jordan AS, White DP, Wellman A, Eckert DJ (2016). Upper airway collapsibility (Pcrit) and pharyngeal dilator muscle activity are sleep stage dependent. Sleep.

[29] Shelton KE, Woodson H, Gay S, Suratt PM (1993). Pharyngeal fat in obstructive sleep apnea. Am Rev Respir Dis.

[30] Schwab RJ, Pasirstein M, Pierson R (2003). Identification of upper airway anatomic risk factors for obstructive sleep apnea with volumetric magnetic resonance imaging. Am J Respir Crit Care Med.

[31] Peppard PE, Young T, Barnet JH, Palta M, Hagen EW, Hla KM (2013). Increased prevalence of sleep-disordered breathing in adults. Am J Epidemiol.

